# Nicotine and smoking in the COVID-19 era

**DOI:** 10.34172/jcvtr.2020.22

**Published:** 2020-05-28

**Authors:** Arezou Tajlil, Samad Ghaffari, Leili Pourafkari, Sina Mashayekhi, Neda Roshanravan

**Affiliations:** ^1^Cardiovascular Research Center, Tabriz University of Medical Sciences, Tabriz, Iran; ^2^University at Buffalo, Buffalo, New York, USA

**Keywords:** COVID-19, Nicotine, Smoking, Coronavirus Disease

## Abstract

***Introduction:*** The knowledge regarding the demographic characteristics of patients with Covid-19 and risk factors distribution is still evolving. Considering the role of cigarette smoking in the pathogenesis of lung diseases and the effect of nicotine on expression of the entry receptor for severe acute respiratory syndrome coronavirus 2 (SARS-CoV-2), it is important to determine the implications of smoking in COVID-19.

***Methods:*** In this brief report, by using the published articles in the literature, we aimed to compare the reported prevalence of smoking in patients with COVID-19 to the prevalence of smoking in the general population of the corresponding report. Binomial tests were conducted and a *P* value of less than 0.05 was considered statistically significant.

***Results:*** Among the screened papers, we found 12 peer-reviewed articles in which epidemiological characteristics of COVID-19 patients, including smoking status, were stated. Based on the descriptive reports of characteristics of COVID-19 patients, we observed a significantly lower proportion of COVID-19 patients with smoking history compared to what is expected, given the population average for each study’s geographic area.

***Conclusion:*** This analysis of available data showed a lower prevalence of smoking in COVID-19 patients in comparison to the regional average. Considering the limitations of the study, the results should be interpreted with great caution and be viewed just as a preliminary report to motivate related basic and clinical researches.

## Introduction

In the ongoing COVID-19 pandemic, protecting individuals with a higher risk of infection and complications is essential. Considering the known role of cigarette smoking in the pathogenesis of lung diseases, understanding the different aspects of COVID-19 in smokers is of great importance.

The tobacco available in cigarette smoke contains nicotine, which interferes with the renin-angiotensin system and notably alters the expression of angiotensin-converting enzyme-2 (ACE-2)^1^, the entry receptor for severe acute respiratory syndrome coronavirus 2 (SARS-CoV-2),^2^ in various organs including lungs.^1^ In addition, nicotine affects the immune cells, exerts anti-inflammatory properties through the cholinergic pathway, and regulates cytokine release.^3^ These effects can potentially play a role in different phases of infection with SARS-CoV-2 and the resulting acute respiratory distress syndrome. Intermittent heating of nasopharynx as a result of inhalation of smoke also may play a role but currently no supporting data is available.

Regarding these facts, using the available articles in the literature, we aimed to compare the reported prevalence of smoking in patients with COVID-19 to the prevalence of smoking in the general population of the corresponding report.^4,5^

## Materials and Methods

A structured literature review in PubMed was conducted on April 28, 2020. We only included papers that were accepted in peer-reviewed journals at the time of literature retrieval. A PubMed search using the terms coronavirus disease 2019 or COVID-19 or COVID or SARS-CoV-2 in the title and/or abstract as well as using the MeSH supplementary concept COVID-19 was performed. Among the screened papers, we found 12 papers in which epidemiological characteristics of COVID-19 patients, including smoking status, were described.^6-17^ We reviewed each report and recorded the data in data collection tool. We used the data for current smokers for our analysis unless a study had no exclusive data for current smokers. For each report, by conducting a binomial test, using GraphPad Prism version 8.0, we compared the observed proportion of smokers in its study sample of patients with COVID-19 to the hypothesized value, based on the prevalence of current smokers in the general population of the corresponding report. The exact binomial *P* value was provided for each test. A *P* value of less than 0.05 was considered statistically significant.

## Results

Table 1 presents the prevalence of smoking in various COVID-19 samples compared to the general population of each report. The reported mean/median age and percentage of males for each study sample were also shown in this table. As presented in Table 1, based on the descriptive reports of characteristics of COVID-19 patients, we observed a significantly lower proportion of COVID-19 patients with smoking history compared to what is expected, given the population average of the study’s geographic area.

**Table 1:** Prevalence of smoking in various COVID-19 samples compared to the general population of each report

**Study**	**Setting**	**Sample** **Size (N)**	**Age Mean (SD)/Mode (IQR)**	**Male** **No. (%)**	**Smokers in Reports of Covid-19 Patients**		**Population Average**	***P*** **value**	**Studied Outcome**	**As Reported in Each Study**		**Reported** ***P*** **value**
					**Observed** **No. (%)**	**95% CI for observed percentage**	**Expected** **No. (%)**			**Smokers in** **Outcome (-)**	**Smokers in Outcome (+)**	
**China**												
Zhou et al^6^	Hospitalized	191	56·0 (46·0–67·0)	119 (62%)	11(6.0%)	3.2 - 10.0	43(22.6%)	<0.001	Death	6 (4%)	5 (9%)	0.21
Guan et al^7^	Hospitalized	1085	47.0 (35.0–58.0)	637 (58.1%)	137(12.6%)	10.8 - 14.7	245(22.6%)	<0.001	Primary Composite End point^*^	120 (11.8%)	17 (25.8%)	-
Liu et al^8^	Hospitalized	78	38 (33, 57)	39 (50.0%)	5(6.4%)	2.8 - 14.1	18(22.6%)	<0.001	Disease Progression	2 (3.0%)	3 (27.3%)	0.018
Guan et al^9^	Hospitalized	1590	48.9 (16.3)	904 (57.3%)	111(7.0%)	5.9 - 8.3	359(22.6%)	<0.001	Death	NA	NA	0.043
Zhang et al^10^	Hospitalized	140	57 (25-87)	71 (50.7)	2(1.4%)	0.3 - 5.1	32(22.6%)	<0.001	Disease Severity	0 (0)	2 (3.4%)	0.170
Huang et al^11^	Hospitalized	41	49·0 (41·0–58·0)	30 (73%)	3(7.0%)	2.6 - 19.5	9(22.6%)	0.010	ICU Care	3 (11%)	0	0·31
Yang et al^12^	Severe	52	59·7 (13·3)	35 (67%)	16(12.8%)	8.0 - 19.8	28(22.6%)	0.004	Death	2 (10%)	0	-
Wang et al^13^	Hospitalized	125	38.7 (13.7)	71 (56.8%)	2(3.0%)	0.7 - 12.9	12(22.6%)	<0.001	Disease Severity	9(9%)	7(28%)	0.027
Tang et al^14^	With ARDS	73	67 (57, 72)	45 (61.6%)	8(11.0%)	5.7 - 20.2	16(22.6%)	0.02	-	-	-	-
Qin et al^15^	Hospitalized	452	58 (47-67)	235 (52.0%)	7(1.5%)	0.7 - 3.2	102(22.6%)	<0.001	Disease Severity	4 (2.4%)	3 (1.0%)	0.267
**U.S.**												
CDC^16^	All Positives	7162	-	-	96(1.3%)	1.1 - 1.6	1225(17.1%)	<0.001	ICU Admission	22 (2%)	5 (1%)	-
**U.S. /New York**												
Goyal et al^17^	Hospitalized	393	62.2 (48.6–73.7)	238 (60.6%)	20(5.1%)	3.3 - 7.7	56(14.2%)	<0.001	Invasive Ventilation	14 (5.3%)	6 (4.6)	-

*Primary composite end point was admission to an intensive care unit (ICU), the use of mechanical ventilation, or death ARDS: acute respiratory distress syndrome, CDC: centers for disease control and prevention, ICU: intensive care unit.

## Discussion

The lower reported prevalence of smoking in COVID-19 in comparison to the regional average is an interesting finding that needs further investigations and clarification. Although not examined in our analysis, the effect of smoking on disease severity, as presented in Table 1, is also inconsistent among studies. In some reports, the prevalence of smoking was higher in COVID-19 patients who experienced an adverse outcome.^8,9,13^ However, other reports did not find a significant association between smoking history and adverse outcomes in COVID-19.^6,10,11,15^ Considering the descriptive nature of the investigated reports with no control group, lack of information about the age groups of smokers in studies, and other confounding factors, our findings should be considered as hypothesis-generating indicating the need for further controlled studies.

Based on earlier studies on SARS-CoV, it is revealed that ACE-2 plays a role in the replication of viral genome and modulation of the immune response in SARS-CoV infected cells, as well.^18^ Adhesion of SARS-CoV to ACE-2 can also ultimately induce a reduction in ACE-2 expression. Since ACE-2 is involved in the production of angiotensin 1-7, which has multiple effects including anti-inflammatory properties, the reduction in the ACE-2 axis activity may exacerbate the progression to the severe acute respiratory syndrome.^19^ In contrast, an increase in ACE-2 axis activity may potentially have protective effects against lung injury.^20^ Cigarette smoking increases ACE-2 expression in lower respiratory system.^18^ While the physiologic function of ACE-2 in the airways is mainly undetermined,^21^ the increased ACE-2 expression as a result of smoking may potentially increase the susceptibility of smokers for SARS-CoV-2 infection. On the other hand, it may theoretically have some anti-inflammatory effects through the upregulation of ACE-2 axis activity as well as modulation of the cholinergic pathway, and regulation of cytokine release. The improper hyperactivation of the NF-kappaB pathway is involved in the latter phase of COVID-19 and is associated with the development of severe acute respiratory syndrome.^22^ Furthermore, exposure to tobacco smoke may inhibit NF-kappaB pathway activation.^23^ While this effect can impair the innate immunity of the smokers,^24^ it may have some implications for reducing the burden of cytokine storm in infected individuals.

Regarding these facts, the possibility of underdiagnosis in smokers and differences in the course of the disease has to be investigated yet. Determining the likelihood of subclinical SARS-CoV-2 infection in smokers as a possible cause of underrepresentation in reported COVID-19 patients has a crucial role in detecting the infected cases and implementing necessary public health measures. Considering the limitations of the study, the findings of this manuscript should be interpreted with great caution and be considered a preliminary report just to motivate researchers in the basic and clinical fields to conduct further related studies.

## Competing interests

The authors declare that they have no competing interests.

## Ethical approval

Not applicable.

## Funding

None.
